# Subclinical and clinical atherosclerosis in rheumatoid arthritis: results from the 3-year, multicentre, prospective, observational GIRRCS (*Gruppo Italiano di Ricerca in Reumatologia Clinica e Sperimentale*) study

**DOI:** 10.1186/s13075-019-1975-y

**Published:** 2019-09-03

**Authors:** Piero Ruscitti, Paola Cipriani, Vasiliki Liakouli, Daniela Iacono, Ilenia Pantano, Domenico Paolo Emanuele Margiotta, Luca Navarini, Giulia Maria Destro Castaniti, Nicola Maruotti, Gerardo Di Scala, Licia Picciariello, Francesco Caso, Sara Bongiovanni, Rosa Daniela Grembiale, Fabiola Atzeni, Raffaele Scarpa, Federico Perosa, Giacomo Emmi, Francesco Paolo Cantatore, Giuliana Guggino, Antonella Afeltra, Francesco Ciccia, Roberto Giacomelli

**Affiliations:** 1 Rheumatology Unit; Department of Biotechnological and Applied Clinical Sciences, University of L’Aquila, delta 6 building, PO box 67100, L’Aquila, Italy; 2 Department of Clinical and Experimental Medicine, Rheumatology Section, University of Campania “Luigi Vanvitelli”, Naples, Italy; 3 Unit of Allergy, Clinical Immunology and Rheumatology, Department of Medicine, Campus Bio-Medico University of Rome, Rome, Italy; 4 Department of Health Promotion, Mother and Child Care, Internal Medicine and Medical Specialties (PROMISE), University of Palermo, Palermo, Italy; 5 Department of Medical and Surgery Sciences, Rheumatology Unit, University of Foggia, Foggia, Italy; 6 Department of Experimental and Clinical Medicine, University of Florence, Florence, Italy; 7 Department of Biomedical Sciences and Human Oncology (DIMO), Rheumatologic and Systemic Autoimmune Diseases Unit, University of Bari Medical School, Bari, Italy; 8 Rheumatology Unit, Department of Clinical Medicine and Surgery, School of Medicine, University of Naples Federico II, Naples, Italy; 9 Unità di Reumatologia, Ospedale L. Sacco, Milan, Italy; 10 Rheumatology Research Unit, Department of Health Sciences, University of Catanzaro ‘Magna Graecia’, Catanzaro, Italy; 11 Rheumatology Unit, Department of Clinical and Experimental Medicine, University of Messina, Messina, Italy

**Keywords:** Rheumatoid arthritis, Atherosclerosis, Cardiovascular diseases, Remission, Type 2 diabetes

## Abstract

**Background:**

Rheumatoid arthritis (RA) is associated with an increased risk of morbidity and mortality, when compared with general population, largely due to enhanced atherosclerotic disease. In this work, we aimed at assessing both occurrence and predictive factors of subclinical and clinical atherosclerosis in RA.

**Methods:**

From January 1, 2015, to December 31, 2015, consecutive participants with RA, admitted to Italian Rheumatology Units, were assessed in the GIRRCS (*Gruppo Italiano di Ricerca in Reumatologia Clinica e Sperimentale*) cohort. After that, those participants were followed up in a 3-year, prospective, observational study, assessing the occurrence of subclinical and clinical atherosclerosis and possible predictive factors. McNemar test was employed to assess the changes in subclinical and clinical atherosclerosis, and regression analyses exploited the ORs for the occurrence of those comorbidities.

**Results:**

We analysed 841 participants, mostly female (82.2%) and with median age of 60 years (range 21–90). The remission was achieved and maintained by 41.8% of participants during the follow-up. We observed an increased rate of subclinical atherosclerosis at the end of follow-up (139 vs 203 participants, *p* < 0.0001), particularly in participants with a disease duration less than 5 years at baseline (70 participants vs 133 participants, *p* < 0.0001). Type 2 diabetes (T2D) (OR 4.50, 95%CI 1.74–11.62, *p* = 0.002), high blood pressure (OR 2.03, 95%CI 1.04–4.14, *p* = 0.042), ACPA (OR 2.36, 95%CI 1.19–4.69, *p* = 0.014) and mean values of CRP during the follow-up (OR 1.07, 95%CI 1.03–1.14, *p* = 0.040) were significantly associated with higher risk of subclinical atherosclerosis. We observed an increased rate of clinical atherosclerosis at the end of follow-up (48 vs 76 participants, *p* < 0.0001). T2D (OR 6.21, 95%CI 2.19–17.71, *p* = 0.001) was associated with a significant risk of clinical atherosclerosis. The achievement and the maintenance of remission reduced the risk of subclinical (OR 0.25, 95%CI 0.11–0.56, *p* = 0.001) and clinical atherosclerosis (OR 0.20, 95%CI 0.09–0.95, *p* = 0.041).

**Conclusions:**

We reported an increased prevalence and incidence of both subclinical and clinical atherosclerosis in 3-year prospectively followed participants, mainly in the subset with a duration of disease less than 5 years. The achievement and the maintenance of remission are associated with a reduction of the risk of subclinical and clinical atherosclerosis. Among “traditional” cardiovascular risk factors, participants with T2D showed a higher risk of clinical and subclinical atherosclerosis.

**Electronic supplementary material:**

The online version of this article (10.1186/s13075-019-1975-y) contains supplementary material, which is available to authorized users.

## Background

Rheumatoid arthritis (RA) is a systemic, inflammatory disease leading to joint damage and to a reduction of quality of life [1, 2]. RA is associated with an increased risk of morbidity and mortality, when compared with general population, largely due to enhanced atherosclerotic disease [3, 4]. Despite the improved long-term joint damage by treatment with synthetic and biologic disease-modifying anti-rheumatic drugs (DMARDs) [5–7], the close relationship between RA and cardiovascular (CV) events has been reported, including myocardial infarction, cerebrovascular accident and congestive heart failure [8, 9]. Furthermore, the prevalence of subclinical atherosclerosis is increased in RA, as shown by studies assessing the rate of carotid artery plaques [10, 11]. Subclinical atherosclerosis may additionally identify those patients with a higher risk to develop CV events [12]. On these bases, multiple lines of research are focused on the development of subclinical and clinical atherosclerosis in RA [13, 14]. This typical clinical phenotype could result from the synergy between the elevated prevalence of “traditional” cardiovascular (CV) risk factors and inflammation [15]. In fact, although the European League Against Rheumatism (EULAR) provided recommendations for the management of CV risk in inflammatory arthritis [16], traditional CV risk factors remain to be underdiagnosed and undertreated in RA, thus contributing to the development of atherosclerotic disease [17]. Concerning the rheumatoid inflammatory process, abundant evidence strongly supports the hypothesis that inflammation could contribute to the pathogenesis of atherosclerotic disease beyond the elevated traditional CV risk factors [18, 19]. Some well-known pathogenic pro-inflammatory RA mediators could play a pivotal role in the development of atherosclerosis, as suggested by both pre-clinical and clinical reports [20, 21]. In this context, it must be pointed out that the evidence deriving from randomised clinical trials does not entirely clarify this issue. In fact, given strict enrolment criteria, the participants, who are usually enrolled, could not fully mirror the real-life scenario, thus decreasing the generalisability of the results [22]. Furthermore, the evaluation of CV burden is mainly obtained from low-quality studies, generally with a retrospective design and affected by different biases impairing both interpretation and generalisation of results. In fact, few prospective studies in this setting have been planned and performed so far. Finally, a comprehensive evaluation of CV burden in the RA could be complex as well as time-consuming, and thus, the identification of biomarkers, accurately reflecting this issue, is still awaited [15, 16, 19, 20, 23].

In this work, we aimed at evaluating both the occurrence and the predictive factors of subclinical and clinical atherosclerosis in participants with RA, in a 3-year, multicentre, prospective, observational study.

## Methods

### Study design

From January 1, 2015, to December 31, 2015, consecutive participants with RA, admitted to Italian Rheumatology Units, were assessed in the GIRRCS (*Gruppo Italiano di Ricerca in Reumatologia Clinica e Sperimentale*) cohort [24]. After that, those participants were followed up in a 3-year, prospective, observational study, assessing the occurrence of subclinical and clinical atherosclerosis and possible predictive factors. The local Ethics Committee (*Comitato Etico Azienda Sanitaria Locale 1 Avezzano/Sulmona/L’Aquila, L’Aquila*, Italy; protocol number 000331/17) approved the study, which was performed according to the Good Clinical Practice guidelines and the Declaration of Helsinki. Informed consent was obtained from each participant for the use of clinical and laboratory data for purposes of the study. In reporting the results, we followed the STROBE checklist (Additional file 1: Table S1).

### Setting

Participants were selected among those attending Rheumatologic Units of GIRRCS, throughout Italy. All the units were characterised by experience in management of RA as well as in observational studies and by high-volume outpatient clinics. Data of participants, who were followed up at least for 3 years, were recorded during the scheduled visits, at baseline, after 12 and 36 months.

### Participants

Participants fulfilling the ACR/EULAR criteria for RA [25, 26] were included in the study.

### Variables to be assessed

The main outcome of the study was the occurrence of subclinical and clinical atherosclerosis in participants with RA, at the end of 3-year prospective follow-up. Subclinical atherosclerosis was defined as the presence of carotid and/or peripheral arteries atherosclerotic lesions detected by ultrasound imaging [27]. Clinical atherosclerosis was defined as the presence of one of the following: myocardial infarction, congestive heart failure, cerebrovascular disease including transitory ischemic attack and/or stroke and clinically relevant peripheral artery disease. The investigators verified history and occurrence of clinical atherosclerosis by review of clinical charts, interview and medical examinations of participants. Following the recent recommendations, about CV disease (CVD) risk assessment to be performed at least once every 5 years [16], we stratified the participants in < 5 and > 5 years of disease duration assessing the main outcomes accordingly. Additionally, by using the JBS3 calculator [28], the 10-year CVD risk was estimated and stratified in 5 categories of risk, < 10% of 10-year CVD risk, > 10% < 20% of 10-year CVD risk, > 20% < 30% of 10-year CVD risk, > 30% < 40% of 10-year CVD risk and > 40% of 10-year CVD risk. Furthermore, predictive factors of these comorbidities were assessed by evaluating two main areas of CV risk factors, namely the traditional and the “RA-related” ones. Among traditional CV risk factors, we assessed gender, age, smoking habit, body mass index (BMI), high cholesterol, metabolic syndrome (MetS), type 2 diabetes (T2D) and high blood pressure (HBP). BMI was calculated according to the standard formula weight (kg)/height (m)^2^. High cholesterol was defined as cholesterol > 240 mg/dl and/or treatment with medications lowering the blood cholesterol levels. MetS, T2D and HBP were defined according to standard criteria and/or treatment with anti-diabetic and/or anti-hypertensive medications [29–31]. Among RA-related risk factors, we assessed the presence of rheumatoid factor (RF) or anti-citrullinated peptide antibodies (ACPA), disease duration, extra-articular features, values of erythrocyte sedimentation rate (ESR, mm/h) and C-reactive protein (CRP), radiographic damage, joint surgery, disease activity and remission. Disease duration was assessed from the first disease symptom at the study beginning. Extra-articular features were defined as reported in previous study [32]. Radiographic damage was defined as the presence of at least one marginal erosion on previously performed hand radiography. Disease Activity Score including 28 joints (DAS28-ESR) was used to assess the disease activity and the remission state (DAS28-ESR < 2.6), as previously defined [33]. ESR was included in the assessment of DAS28 in order to maintain the independence of CRP, a well-established CV risk factor in the general population, for purpose of data analysis. Participants in remission were defined as those reaching after 12 months and maintaining after 36 months a value of DAS28-ESR < 2.6.

We recorded the administered therapeutic strategies during the follow-up. For those participants who underwent sequential treatment with synthetic or biologic DMARDs, we assigned the treatment category according to the medication to which the participant was exposed for a longer period. Corticosteroids (CCSs) treatment was codified in categories, high dosage and low dosage, as previously identified [34]. We defined participants treated with high dosage of CCSs as those taking > 7.5 mg prednisone-equivalent for 3 months or more during the observation period, whereas participants treated with low dosage of CCSs as those taking ≤ 7.5 mg prednisone-equivalent. We also recorded the use of aspirin (ASA) during the follow-up.

### Data sources

Relevant data were collected at study beginning and reassessed after 12 and 36 months, during the scheduled visits for each involved participant by an extensive clinical history.

### Bias

Considering the observational design, our study could be subjected to a number of possible biases. We tried to minimise the main methodological problems by a careful definition of each variable to be assessed. Furthermore, participants with significant missing data, which were considered to be meaningful for the analyses, were removed. Specifically, participants with one or more missing data in the main outcomes were removed from the analyses.

### Study size

We would provide a “real-life” estimation of the occurrence of subclinical and clinical atherosclerosis in consecutive participants with RA in Italian Rheumatology Units. From January 1, 2015, to December 31, 2015, consecutive participants with RA, admitted to Italian Rheumatology Units, were assessed and followed up in a 3-year, prospective, observational study.

### Statistical methods

Statistics firstly provided descriptive analysis of the data. Normally distributed continuous variables were expressed as mean ± standard deviation (SD), otherwise as median and range interquartile, as appropriate. McNemar test was employed to assess the changes in subclinical and clinical atherosclerosis comparing the beginning and the end of follow-up and different subsets of participants. Incident cases were reported as incidence proportion and incidence rate per 1000 person-years at risk. Regression analyses exploited the ORs for the occurrence of those comorbidities, considering data at 36 months. The purposeful selection process of covariates started by a univariate analysis of each variable; any variable having a significant univariate test was selected as a possible candidate for the multivariate analyses. Conversely, covariates were removed from the models if non-significant. At the end of this process of deleting and refitting, the multivariate models were built and OR estimations of significant associations with subclinical and clinical atherosclerosis were provided. Multicollinearity was evaluated by using the variance inflation factor (VIF) before entering each variable in regression models. Participants characterised by missing data in main outcomes were excluded from the analysis. Two-sided *P* values < 0.05 were considered statistically significant. The Statistics Package for Social Sciences (SPSS for Windows, version 17.0, SPSS Inc., Chicago, IL, USA) was used for all analyses.

## Results

### Participants and descriptive data

After assessment of 886 participants, 841 participants with 3 years of prospective follow-up were analysed. In the present evaluation, only participants fully followed up for 3 years were assessed, follow-up at 12 and 36 months was performed in all these participants. The participants, who were excluded, were characterised by missing data in main outcomes. Baseline characteristics of participants are described elsewhere [24]. Briefly, assessed participants were mostly female (82.2%), median age of 60 years (range 21–90), median disease duration of 8.20 years (range 0.1–35), 73.1% displayed the positivity for RF and/or for ACPA. During the follow-up, 72.8% of the participants were treated with CCSs (mainly at low dosage, 60.0% of participants), 85.1% with methotrexate (MTX), and 61.5% with biologic DMARDs. Concerning the clinical response, we observed that 41.8% of participants reached and maintained the remission during the follow-up. Regarding traditional CV risk factors, 31.6% of the participants reported smoking habit, 49.3% were affected by HBP, 32.1% by high levels of cholesterol, 22.3% by MetS and 12.1% by T2D, as shown in Table 1.

**Table 1:** Descriptive statistics

Clinical variables	
Participants, number	841
Demographic characteristics	
Age (841 participants), median (range)	60 years (21–90)
Female gender (841 participants), *n* (%)	691 (82.2%)
RA-related features	
RF and/or ACPA (841 participants), *n* (%)	615 (73.1%)
Disease duration (834 participants), median (range)	8.2 years (0.1–35)
Extra-articular features (840 participants), *n* (%)	138 (16.4%)
Radiographic damage (814 participants), *n* (%)	383 (47.1%)
Joint surgery (841 participants), *n* (%)	101 (12.4%)
Maintenance of remission (836 participants), *n* (%)	349 (41.8%)
CRP (833 participants), mean ± SD	4.31 ± 3.62 mg/L
Traditional CV risk factors	
BMI (829 participants), mean ± SD	27.01 ± 4.02
HBP (811 participants), *n* (%)	400 (49.3%)
High cholesterol (798 participants), *n* (%)	256 (32.1%)
Smoking habit (836 participants), *n* (%)	264 (31.6%)
MetS (807 participants), *n* (%)	180 (22.3%)
T2D (811 participants), *n* (%)	98 (12.1%)
Therapies	
ASA (838 participants), *n* (%)	320 (38.2%)
CCS (841 participants), *n* (%)	612 (72.8%)
CCSs low dosage (841 participants), *n* (%)	504 (60.0%)
MTX (841 participants), *n* (%)	716 (85.1%)
HCQ (813 participants), *n* (%)	231 (28.5%)
LEF (841 participants), *n* (%)	186 (22.1%)
SSZ (841 participants), *n* (%)	113 (13.4%)
Biologic DMARDs (841 participants), *n* (%)	517 (61.5%)
TNFi (841 participants), *n* (%)	308 (36.6%)
Non TNFi (841 participants), *n* (%)	209 (24.9%)

Participants with missing data, *n* (%): disease duration, 7 (0.8%); extra-articular features, 1 (0.1%); radiographic damage, 27 (3.2%); maintenance of remission, 5 (0.6%); CRP, 8 (0.9%); BMI, 12 (1.4%); high cholesterol, 43 (5.1%); HBP, 30 (3.6%); smoking habit, 5 (0.6%); MetS, 34 (4.0%); T2D, 30 (3.6%); ASA, 3 (0.3%); HCQ, 28 (3.3%)

*Abbreviations*: *RA* rheumatoid arthritis, *RF* rheumatoid factor, *ACPA* Anti-citrullinated protein antibodies, *CRP* mean values of C reactive protein during the follow-up, *SD* standard deviation, *CV* cardiovascular, *BMI* mean body mass index during the follow-up, *MetS* metabolic syndrome, *HBP* high blood pressure, *T2D* type 2 diabetes, *ASA* acetylsalicylic acid, aspirin, *CCSs* corticosteroids, *MTX* methotrexate, *HCQ* hydroxychloroquine, *SSZ* sulfasalazine, *LEF* leflunomide, *TNFi* tumour necrosis factor inhibitor

### Occurrence of subclinical atherosclerosis

**Fig. 1:**
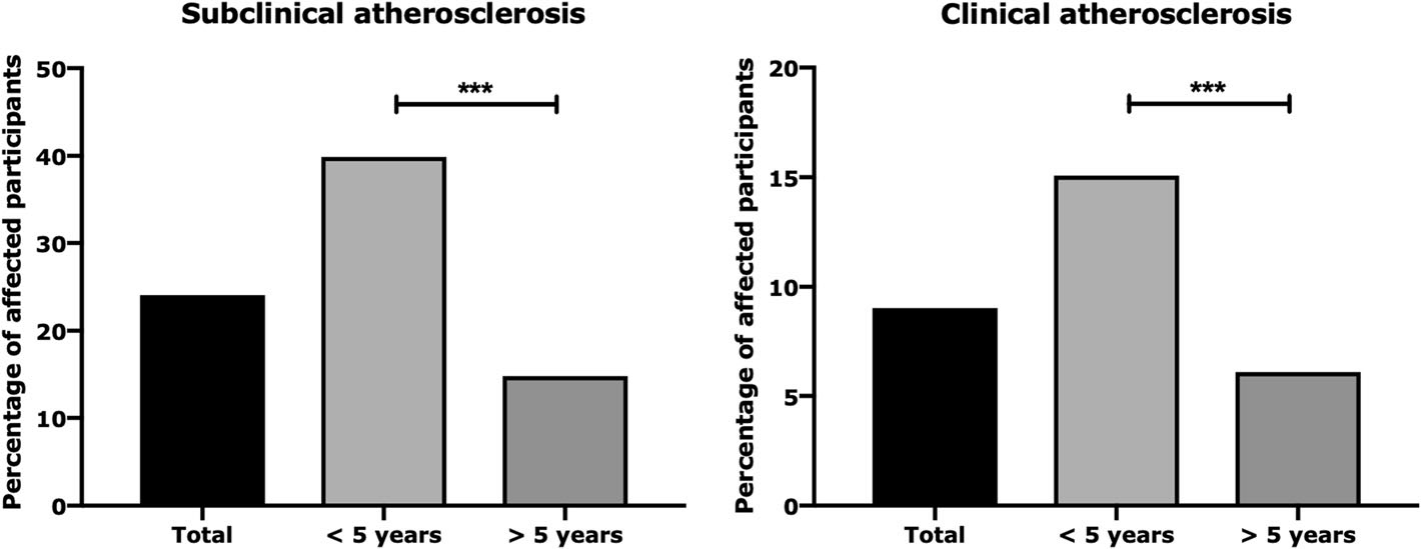
Prevalence of subclinical and clinical atherosclerosis. Analysing the prevalence according to the duration of the disease, we stratified the participants based on duration of disease < 5 years or duration of disease > 5 years. In participants with duration of disease < 5 years, we recorded an increased rate of both subclinical and clinical atherosclerosis when compared with participants with duration of disease > 5 years. ***p < 0.0001

We recorded that 24.1% [21.3–26.7] of participants were defined as having subclinical atherosclerosis at the end of follow-up, an increased rate when compared with the beginning of the study (139 participants vs 203 participants, *p* < 0.0001). All participants included in the present evaluation underwent ultrasound of peripheral arteries, which was performed at baseline, after 12 and 36 months. Analysing the incident cases of subclinical atherosclerosis, we estimated an incidence proportion of 10.1% [8.0–12.0] and, considering over 2010 person-years, an incidence rate of 31.8 × 1000 [24.8–38.8] person-years. Furthermore, we analysed these results according to the duration of the disease, stratifying the participants based on duration of disease < 5 years or duration of disease > 5 years (Fig. 1). In participants with duration of disease < 5 years, we recorded an increased rate of subclinical atherosclerosis when compared with participants with duration of disease > 5 years (70 participants vs 133 participants, *p* < 0.0001). Analysing the incident cases of subclinical atherosclerosis in these subsets, we estimated an incidence proportion of 20.7% [18.1–23.3] and, considering over 610 person-years, an incidence rate of 77.1 × 1000 person-years [56.6–97.6] in participants with duration of disease < 5 years. Conversely, in participants with duration of disease > 5 years, we estimated an incidence proportion of 5.6% [3.4–7.8] and, considering over 1149 person-years, an incidence rate of 19.1 × 1000 person-years [8.4–29.8] in participants with duration of disease > 5 years.

### Predictive factors of subclinical atherosclerosis

A logistic regression model was built in 697 participants to evaluate the possible predictive role of selected variables (age, male gender, HBP, T2D, ACPA, mean values of CRP during the follow-up, remission) on the likelihood of having subclinical atherosclerosis, after 36 months. The participants, who were excluded, were characterised by baseline evidence of subclinical and/or clinical atherosclerosis. The analysis showed that T2D, HBP, ACPA, remission and mean values of CRP during the follow-up were independently associated with subclinical atherosclerosis. Participants with comorbid T2D (OR 4.50, 95%CI 1.74–11.62, *p* = 0.002) and with comorbid HBP (OR 2.03, 95%CI 1.04–4.14, *p* = 0.042) were significantly associated with a higher risk of having subclinical atherosclerosis. The presence of ACPA (OR 2.36, 95%CI 1.19–4.69, *p* = 0.014) and mean values of CRP during the follow-up (OR 1.07, 95%CI 1.03–1.14, *p* = 0.040) were also significantly associated with a higher risk of that comorbidity. Of interest, the participants reaching and maintaining remission were significantly associated with a reduced risk of having subclinical atherosclerosis (OR 0.25, 95%CI 0.11–0.56, *p* = 0.001), as shown in Table 2. The logistic regression model was statistically significant (χ^2^ = 13.09, *p* < 0.0001). Regarding the possible predictive role of treatment on subclinical atherosclerosis, we did not retrieve any significant result concerning the possible predictive role of ASA, MTX, HCQ, low dosage CCSs, high dosage CCSs, TNFi and non-TNFi.

**Table 2:** Regression analyses assessing predictive factors of subclinical atherosclerosis

Clinical variables	OR	95%CI	*p*
Univariate analyses			
Age	1.01	0.99–1.02	0.10
Male gender	1.18	0.79–1.76	0.42
RF	1.15	0.81–1.65	0.43
ACPA	1.40	1.01–1.94	*0.042*
Disease duration	1.00	0.98–1.02	0.99
Extra-articular features	1.31	0.74–1.72	0.56
Radiographic damage	0.85	0.61–1.16	0.30
Joint surgery	1.32	0.83–2.08	0.23
Maintenance of remission	0.65	0.47–0.91	*0.014*
CRP	1.04	1.02–1.07	*0.002*
BMI	1.02	0.89–1.08	0.52
HBP	8.79	5.87–13.18	*< 0.0001*
High cholesterol	2.32	0.89–5.53	0.89
Smoking habit	0.99	0.71–1.40	0.99
MetS	3.99	2.83–5.64	*< 0.0001*
T2D	3.16	2.04–4.88	*< 0.0001*
ASA	2.59	0.98–4.13	0.08
CCS	1.36	0.68–2.04	0.78
CCSs low dosage	1.25	0.87–1.78	0.22
MTX	1.67	0.98–2.75	0.08
HCQ	0.62	0.42–1.01	0.09
LEF	0.73	0.49–1.09	0.13
SSZ	0.98	0.61–1.57	0.95
Biologic DMARDs	0.58	0.42–1.01	0.07
TNFi	0.99	0.71–1.37	0.95
Non-TNFi	0.67	0.24–1.02	0.12
Multivariate analysis			
Age	1.20	0.99–1.05	0.13
Male gender	1.29	0.55–3.04	0.55
HBP	2.03	1.04–4.14	*0.042*
T2D	4.50	1.74–11.62	*0.002*
ACPA	2.36	1.19–4.69	*0.014*
CRP	1.07	1.03–1.14	*0.040*
Remission	0.25	0.11–0.56	*0.001*

Italicised values are statistically significant (*p* < 0.05)

*Abbreviations*: *RF* rheumatoid factor, *ACPA* Anti-citrullinated protein antibodies, *CRP* mean values of C reactive protein during the follow-up, *BMI* mean body mass index during the follow-up, *MetS* metabolic syndrome, *HBP* high blood pressure, *T2D* type 2 diabetes, *ASA* acetylsalicylic acid, aspirin, *CCSs* corticosteroids, *MTX* methotrexate, *HCQ* hydroxychloroquine, *SSZ* sulfasalazine, *LEF* leflunomide, *TNFi* tumour necrosis factor inhibitor

### Occurrence of clinical atherosclerosis

We observed that 9.0% [8.8–9.2] of participants were defined as having clinical atherosclerosis at the end of the follow-up, an increased rate when compared with the beginning of the study (48 participants vs 76 participants, *p* < 0.0001). Analysing the incident cases of clinical atherosclerosis, we estimated an incidence proportion of 3.6% [2.4–4.8] and, considering over 2337 person-years, an incidence rate of 12.0 × 1000 person-years [8.0–16.0]. Furthermore, we analysed these results according to the duration of the disease, stratifying the participants based on duration of disease < 5 years or duration of disease > 5 years (Fig. 1). In participants with duration of disease < 5 years, we recorded an increased rate of clinical atherosclerosis when compared with participants with duration of disease > 5 years (27 participants vs 49 participants, *p* < 0.0001). Analysing the incident cases of clinical atherosclerosis in these subsets, we estimated an incidence proportion of 19.5% [14.8–24.1] and, considering over 832 person-years, an incidence rate of 32.4 × 1000 person-years [20.4–44.4] in participants with duration of disease < 5 years. Conversely, in participants with duration of disease > 5 years, we estimated an incidence proportion of 0.5% [0.2–0.8] and, considering over 1248 person-years, an incidence rate of 1.6 × 1000 person-years [0.6–3.8] in participants with duration of disease > 5 years.

In 697 participants without baseline evidence of subclinical and clinical atherosclerosis, the 10-year CVD risk was estimated. We retrieved that 267 participants (38.4%) displayed < 10% of 10-year CVD risk, 249 (40.0%) < 10% < 20% of 10-year CVD risk, 86 (12.3%) > 20% < 30% of 10-year CVD risk, 43 (6.2%) > 30% < 40% of 10-year CVD risk and 22 (3.1%) > 40% of 10-year CVD risk.

### Predictive factors of clinical atherosclerosis

A logistic regression model was built in 697 participants to evaluate the possible predictive role of selected variables (age, male gender, HBP, T2D, remission) on the likelihood of having clinical atherosclerosis. The participants, who were excluded, were characterised by baseline evidence of subclinical and/or clinical atherosclerosis. The analysis showed that T2D and remission were independently associated with subclinical atherosclerosis. Participants with comorbid T2D (OR 6.21, 95%CI 2.19–17.71, *p* = 0.001) were associated with a higher risk of having clinical atherosclerosis. Conversely, the participants reaching and maintaining remission were associated with a reduced risk of clinical atherosclerosis (OR 0.20, 95%CI 0.09–0.95, *p* = 0.041), as shown in Table 3. The logistic regression model was statistically significant (*χ*^*2*^ = 6.37, *p* = 0.012). As reported for subclinical atherosclerosis, we did not retrieve any significant result concerning the possible predictive role of ASA, MTX, HCQ, low dosage CCSs, high dosage CCSs, TNFi and non-TNFi on clinical atherosclerosis.

**Table 3:** Regression analyses assessing predictive factors of clinical atherosclerosis

Clinical variables	OR	95%CI	*p*
Univariate analyses			
Age	1.01	0.99–1.03	0.30
Male gender	1.14	0.63–2.08	0.65
RF	1.33	0.77–2.32	0.31
ACPA	0.73	0.45–1.28	0.20
Disease duration	0.99	0.95–1.02	0.58
Extra-articular features	1.67	0.94–2.93	0.08
Radiographic damage	089	0.55–1.43	0.63
Joint surgery	0.82	0.38–1.76	0.61
Maintenance of remission	0.55	0.34–0.91	*0.20*
CRP	1.02	0.99–1.05	0.14
BMI	1.05	0.99–1.08	0.11
HBP	10.69	5.06–22.59	*< 0.0001*
High cholesterol	2.47	0.56–5.9	0.18
Smoking habit	0.80	0.47–1.35	0.40
MetS	6.27	3.79–10.37	*< 0.0001*
T2D	2.64	1.48–4.71	*0.001*
ASA	1.11	0.78–2.01	0.85
CCS	1.44	0.81	2.56
CCSs low dosage	1.91	0.89–3.46	0.09
MTX	1.31	0.64–2.72	0.45
HCQ	0.61	0.34–1.08	0.09
LEF	0.51	0.25–1.07	0.07
SSZ	0.53	0.23–1.24	0.14
Biologic DMARDs	0.67	0.29–1.36	0.35
TNFi	0.55	0.32–1.01	0.07
Non-TNFi	0.59	0.31–1.02	0.09
Multivariate analysis			
Age	1.01	0.99–1.05	0.44
Male gender	0.62	0.22–1.73	0.36
HBP	1.85	0.69–4.91	0.21
T2D	6.21	2.19–17.71	*0.001*
Remission	0.20	0.09–0.95	*0.041*

Italicised values are statistically significant (*p* < 0.05)

*Abbreviations*: *RF* rheumatoid factor, *ACPA* Anti-citrullinated protein antibodies, *CRP* mean values of C reactive protein during the follow-up, *BMI* mean body mass index during the follow-up, *MetS* metabolic syndrome, *HBP* high blood pressure, *T2D* type 2 diabetes, *ASA* acetylsalicylic acid, aspirin, *CCSs* corticosteroids, *MTX* methotrexate, *HCQ* hydroxychloroquine, *SSZ* sulfasalazine, *LEF* leflunomide, *TNFi* tumour necrosis factor inhibitor

## Discussion

Although the increased CV risk has been established since many years in RA, only few prospective studies in this setting have been planned and performed. Thus, we designed a 3-year prospective study, enrolling a large cohort of participants, in order to provide a real-life estimation of the CV burden in RA. We reported an increased prevalence and incidence of both subclinical and clinical atherosclerosis in prospectively followed participants, mainly in the subset with a duration of disease less than 5 years, suggesting the role of active inflammatory process. We also showed that the occurrence of subclinical and clinical atherosclerosis derives from a synergy between both RA-related and traditional CV risk factors, pointing out the need of a strict management of both systemic inflammation and CV risk factors to improve the long-term outcome of RA.

In our study, we observed a lower occurrence of subclinical and clinical atherosclerosis than reported in available meta-analyses on these topics [3, 4, 10]. However, our results showed a noteworthy increased prevalence and incidence of both subclinical and clinical atherosclerosis in participants with a duration of disease less than 5 years. The decreased prevalence of both subclinical and clinical atherosclerosis observed in our participants with longer disease duration cannot be attributed to deaths or participants lost to follow-up; due to the specific design of our study, we analysed only participants fully completing 3 years of prospective follow-up. Furthermore, these results could suggest that the increased risk of subclinical and clinical atherosclerosis is already present in the early stages of RA [35, 36], and not only related to the reported accrual CV damage [3, 4, 10]. In this context, patients with RA could be more prone to plaque instability and rupture, in addition to accelerated atherosclerosis [9]. The inflammatory process could contribute more specifically to more severe acute coronary syndromes and strokes [37], which may be more strongly associated with the presence and severity of local or systemic inflammation than with disease duration [38]. In addition, some reversals of the vascular damage could be achieved after anti-inflammatory therapies [39, 40], suggesting the long-term improvement of CV burden observed in our study. However, considering the evidence of some conflicting results concerning the role of duration of disease on accelerated atherosclerosis in RA [41], further specifically designed studies are necessary to entirely elucidate this issue of CV burden in early stages of the disease.

Analysing predictive factors of occurrence, we clearly showed that remission significantly reduced the risk of both subclinical and clinical atherosclerosis. We observed that participants reaching and maintaining the remission during the follow-up experienced a lesser risk of having these comorbidities. Our data suggest that the inflammatory process may strongly contribute in enhancing the CV risk [14, 15, 42] and the remission ought to be considered the pivotal goal for CV risk management in patients with RA [43]. In fact, a treat-to-target intervention, aiming at remission, showed to significantly reduce the occurrence of clinical and subclinical atherosclerosis in a clinical trial [16, 44]. As suggested by our data, the consequent decline of CV burden in RA could be attributable to this better disease management [13, 45]. In fact, the delay of diagnosis and therapy of RA could enhance the clinical atherosclerosis, supporting the importance of early recognition and treatment of these patients [46]. Assessing further predictive factors among the RA-related CV risk factors, we reported an association between ACPA positivity and elevated CRP levels with subclinical atherosclerosis. In fact, patients affected by a seropositive disease with high inflammatory burden experience an accelerated atherosclerosis [47]. Our prospective study confirms previous paper reporting the ACPA positivity as a risk factor for development of atherosclerosis in RA, independently of traditional ones [48]. It has been also suggested that ACPA could impair the resolution of inflammation within the atherosclerotic plaque, enhancing the evolution and destabilisation of the lesions [19, 49]. In addition, ACPA positivity could correlate with vascular calcifications in RA, a further marker of accelerated atherosclerosis [50].

Analysing traditional CV risk factors, we observed that participants with comorbid T2D were at higher risk of both subclinical and clinical atherosclerosis. Insulin resistance and T2D were shown as being highly prevalent in RA and to enhance the CV burden [51, 52]. Furthermore, T2D and RA could share pathogenic inflammatory pathways suggesting possible common therapeutic targets [53–55]. We also observed that participants with comorbid HBP were associated with enhanced risk of subclinical atherosclerosis. In fact, HBP was reported as being highly prevalent in RA, and it is considered one of the most important predictor of atherosclerosis in rheumatic diseases [56]. Conversely, in our analyses, some well-known traditional CV risk factors were not associated with subclinical and clinical atherosclerosis, including BMI, cholesterol and smoking habit. Our study confirms what already reported about some RA paradoxes in the analysis of CV risk. In fact, the rheumatoid sarcopenia, altering the body composition, impairs the predictive role of BMI on atherosclerotic disease, and the qualitative changes of lipoproteins induced by the rheumatoid process cannot be routinely assessed by the quantitative tests, thus limiting the predictive role of dyslipidaemia in these participants [57, 58]. Finally, the “smoking paradox” was described in RA due to an index event bias, since smoking habit is associated with RA and with its associated comorbidities [59].

The results of this study did not show the predictive role of administered therapies on the main outcomes. In fact, based on the real-life design of the study, the therapies were not systematically administered, and the choice of medications was left to the physicians, with the consequent risk of a “confounding by indication” bias, due to the possibility that more intensive treatment could be administered to those participants affected by a more aggressive disease. In this context, the lack of randomised controlled trials, specifically designed to evaluate the effect of different drugs in controlling the insurgence of atherosclerotic disease in RA, could impair the possibility to reach definitive conclusions.

Our study could be affected by different limitations, reducing the external validity of the results and suggesting a cautious interpretation. Despite providing an insight into CV risk associated with RA, the lack of a control group avoided to quantify the relative risk of new-onset subclinical and clinical atherosclerosis when compared with matched participants from general population. Furthermore, the lack of data regarding aortic atherosclerosis, thoracic or abdominal aortic aneurysm and angina pectoris could be considered a further limitation of the present work. In addition, the original study design did not allow to fully ascertain the role of therapeutic strategies on subclinical and clinical atherosclerosis. Further studies, specifically designed and adequately powered, are needed to fully elucidate the CV burden in RA and the best therapeutic strategy of those comorbidities and to confirm the results of the more recent diagnostic techniques and biomarkers in CV assessment in RA [60–62]. Finally, the management of missing data by listwise deletion, removing all data for an observation that has one or more missing values, could also be disadvantageous than imputation methods, such as last observation carried forward. In fact, although we considered of not having inadvertently removed a class of participants and that our sample could be large enough to drop data without substantial loss of statistical power, the assumptions of “missing completely at random” could be typically difficult to support, producing possible biased estimates.

## Conclusions

In conclusion, we reported an increased prevalence and incidence of both subclinical and clinical atherosclerosis in prospectively followed participants, mainly in the subset with a duration of disease less than 5 years. We observed that achievement and maintenance of remission is associated with a reduction of subclinical and clinical atherosclerosis in prospectively followed participants. Among traditional CV risk factors, T2D was significantly associated with both subclinical and clinical atherosclerosis, while about other traditional CV risk factors the RA-specific paradoxes may limit the role of tests assessing the lipid profile, smoking habit and BMI. Taking together, our data support the need of a multi-expertise management of RA, counteracting the synergy between the systemic inflammatory process and the traditional CV risk factors in reducing the CV burden and, thus, improving the long-term outcome of patients with RA.

## Additional file

Additional file 1: STROBE 2007 (v4) checklist of items to be included in reports of observational studies in epidemiology* (DOC 96 kb)

## Data Availability

All data relevant to the study are included in the article or uploaded as supplementary information.

## Abbreviations

RA: Rheumatoid arthritis
DMARDs: Disease-modifying anti-rheumatic drugs
CV: Cardiovascular
EULAR: European League Against Rheumatism
GIRRCS: *Gruppo Italiano di Ricerca in Reumatologia Clinica e Sperimentale*
BMI: Body mass index
MetS: Metabolic syndrome
T2D: Type 2 diabetes
HBP: High blood pressure
RF: Rheumatoid factor
ACPA: Anti-citrullinated peptide antibodies
ESR: Erythrocyte sedimentation rate
CRP: C-reactive protein
DAS28: Disease Activity Score including 28 joints
CCSs: Corticosteroids
ASA: Aspirin
SD: Standard deviation
VIF: Variance inflation factor
MTX: Methotrexate
HCQ: Hydroxychloroquine
TNFi: Tumour necrosis factor inhibitor
